# Synthesis of Dendritic ZSM-5 Zeolite through Micellar Templating Controlled by the Amphiphilic Organosilane Chain Length

**DOI:** 10.1021/acs.cgd.3c00326

**Published:** 2023-06-28

**Authors:** María del Mar Alonso-Doncel, Elena A. Giner, Daniel de la Calle, Jennifer Cueto, Patricia Horcajada, Rafael A. García-Muñoz, David P. Serrano

**Affiliations:** † Thermochemical Processes Unit, IMDEA Energy Institute, Avda. Ramón de la Sagra 3, E28935 Móstoles, Madrid, Spain; ‡ Advanced Porous Materials Unit, IMDEA Energy Institute, Avda. Ramón de la Sagra 3, E28935 Móstoles, Madrid, Spain; § Chemical and Environmental Engineering Group, Rey Juan Carlos University, c/Tulipán s/n, E28933 Móstoles, Madrid, Spain

## Abstract

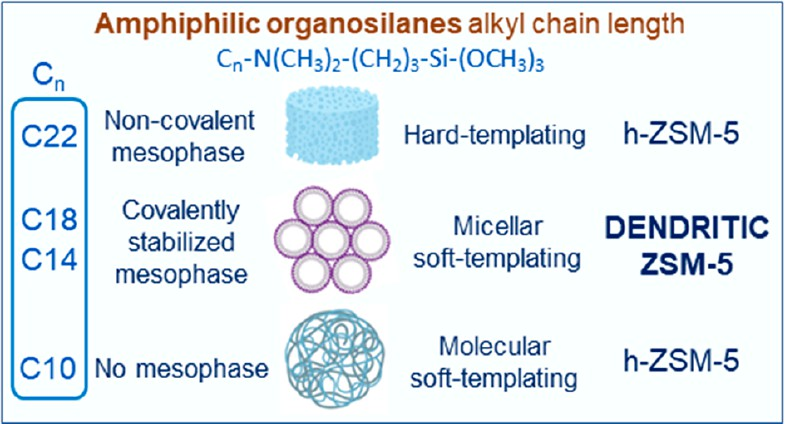

The synthesis of ZSM-5 zeolites by hydrothermal crystallization of protozeolitic nanounits functionalized with amphiphilic organosilanes of different chain length (C_*n*_-N(CH_3_)_2_-(CH_2_)_3_-Si-(OCH_3_)_3_, *n* = 10, 14, 18 and 22) has been investigated. Well-developed dendritic nanoarchitectures were achieved when using C14 and C18 organosilanes, exhibiting a radial and branched pattern of zeolitic nanounits aggregates. In contrast, although C10 and C22 organosilanes led to materials with hierarchical porosity, they lack of dendritic features. These differences have been linked to the formation of an amorphous mesophase at the gel preparation stage for the C14 and C18 samples, in which the surfactant micelles are covalently connected with the protozeolitic nanounits through siloxane bonds. The presence of the dendritic nanostructure positively impacts both the textural and catalytic properties of ZSM-5 zeolite. Thus, ZSM-5 (C14) and ZSM-5 (C18) samples exhibit the largest contribution of mesoporosity in terms of both surface area and pore volume. On the other hand, when tested as catalysts in the aldol condensation of furfural with cyclopentanone, which is an interesting reaction for the production of sustainable jet fuels, the highest catalytic activity is attained over the dendritic ZSM-5 materials due to their remarkable accessibility and balanced Brønsted/Lewis acidity.

## Abstract 2

Hierarchical ZSM-5 zeolite nanoarchitecture can be modulated by varying the chain length of amphiphilic organosilanes. The formation of a covalently stabilized mesophase is a necessary condition for obtaining dendritic ZSM-5 zeolites through a micellar soft-templating mechanism. This mesophase is formed in the synthesis gel when employing organosilanes with a suitable hydrophobicity.

## 1 Introduction

Accessibility issues in zeolites have attracted great research activity due to the severe steric and transport limitations imposed by the small size of zeolitic micropores, usually below 1 nm. Several strategies have been proposed for overcoming steric and diffusional constraints in zeolites. The reduction of the zeolite crystal size (nanozeolites) is one of the most widely studied alternatives.^1−3^ Likewise, another interesting strategy consists of the introduction of a secondary porosity that may lie in the supermicro-, meso-, and macropore ranges (hierarchical zeolites), which has arisen a huge amount of interest during the past 15 years.^4−7^ In comparison with conventional microporous zeolites, hierarchical ones have shown a number of relevant advantages derived from their enhanced accessibility. Thus, the presence of a large share of mesopore surface area, which may represent up to 50% of the overall surface area, has made possible their use as efficient catalysts in a large variety of chemical transformations involving bulky reactants or products, unable to enter or leave the zeolite micropores.^8^ Moreover, even for reactions taking place inside the zeolite microporosity, an important enhancement of the catalytic activity has been derived from the presence of the secondary mesoporosity as a consequence of the shortening of the intracrystalline diffusional pathway.^4,9^ In the same way, the zeolite catalyst lifetime has often been significantly extended over hierarchical zeolitic materials due to a lower deactivating effect of the coke deposits. A typical example is the methanol conversion into hydrocarbons, in which the ZSM-5 zeolite lifetime has sharply improved when using samples with hierarchical porosity.^10^

On the other hand, generation of mesopores in zeolites may also induce important changes in some of the physicochemical properties inherent to this class of materials, like shape-selectivity and acidity. In this sense, chemical reactions occurring over active sites located on the mesopore surface area are expected to take place with no or little shape-selectivity effects. This may provoke strong variations in the product distribution regarding the transformations occurring under confinement within the zeolite microporosity. Likewise, when comparing conventional zeolites with their hierarchical counterparts, significant changes have also been observed in the nature and strength of the acid sites. Thus, ZSM-5 zeolites with hierarchical porosity usually exhibit an important fraction of Lewis acid sites and a reduced concentration of Brønsted ones in comparison with conventional Al-containing MFI materials.^11,12^ This may decrease the catalytic activity in some transformations, like cracking and aromatization reactions but, in contrast, may promote reaction pathways involving Lewis acid sites, such as Meerwein–Ponndorf–Verley reduction or Friedel–Crafts acylation.^11,13^

Soft-templating methods are one of the most employed strategies for obtaining hierarchical zeolitic materials due to the versatility of the procedures so employed and the variety of mesopore generating agents that can be introduced in the synthesis, which include different types of organosilanes,^12,14^ diquaternary-ammonium surfactants^15−17^ and polymers,^18−20^ among others. Using these templates and adjusting the synthetic conditions has made possible to obtain hierarchical zeolites with a variety of morphologies and configurations, such as nanocrystals aggregates, nanosponges, unilamellar and multilamellar structures.^21,22^

In a recent work,^23^ we have reported that the functionalization of MFI embryos with an amphiphilic organosilane results in the formation of a hierarchical ZSM-5 zeolite showing a singular dendritic nanoarchitecture. The samples thus obtained exhibit a radial pattern of branched nanounits, showing enhanced textural properties and a highly interconnected trimodal porosity. This is a remarkable achievement since just rare examples can be found so far in the literature on the preparation of zeolites with dendritic features.^24^ Moreover, considering the current high interest in the synthesis and application of dendritic materials based on amorphous silica,^25−27^ it can be envisaged a great potential of dendritic zeolites for the development of novel catalysts, adsorbents and/or carriers with advanced properties.

In this context, this work reports the strong effect of the length of the amphiphilic organosilane, employed for the functionalization of MFI protozeolitic nanounits, on the generation of a dendritic nanostructure in ZSM-5 zeolites upon hydrothermal crystallization. In addition, the resulting materials have been investigated here as catalysts for aldol condensation of furfural and cyclopentanone, demonstrating the superior performance of the dendritic nanoarchitectures in comparison with those of less organized hierarchical porosities. Thus, furfural and cyclopentanone, which could be obtained by lignocellulosic biomass pyrolysis, are converted into larger molecules with 10 and 15 carbons via aldol condensation,^28^ which in turn could be used for the production of advanced biofuels.

## 2 Experimental Procedures

### 2.1 Organosilane Synthesis and Characterization

All reagents were obtained from commercial sources and used without further purification unless noted otherwise. ^1^H and ^13^C NMR spectra were recorded with a Bruker Avance NEO 400 MHz (^1^H NMR) spectrometer, using CDCl_3_ as the solvent with the residual solvent signal as the internal reference (CDCl_3_, 7.27 and 77.16 ppm).

Amphiphilic organosilanes of different chain lengths (C_*n*_-N(CH_3_)_2_-(CH_2_)_3_-Si-(OCH_3_)_3_, *n* = 10, 14, 18 and 22) were employed, being named as C10, C14, C18 and C22, respectively. The C14 (tetradecyldimethyl(3-trimethoxysilylpropyl)ammonium chloride; Gelest; 50% in methanol) and C18 (dimethyloctadecyl[3-(trimethoxysilyl)propyl]ammonium chloride; Aldrich, 42% in methanol) organosilanes are commercially available and were used without any purification.

#### 2.1.1 Synthesis of C10

In a flamed flask, 1-chlorodecane (2 g; 6.78 mmol) was dissolved in 40 mL of anhydrous methanol under an inert atmosphere. (*N*,*N*-Dimethylaminopropyl)trimethoxysilane (1.6 mL, 6.78 mmol) was added, and then this mixture was stirred under reflux for 48 h. When the reaction was finished, methanol was evaporated under vacuum, and the residue was washed with hexane. The product was obtained as a pale yellow oil with 84.83% yield (2.93 g): ^1^H NMR (400 MHz, CDCl_3_) δ 3.59–3.50 (m, 12H), 3.50–3.46 (m, 2H), 1.83–1.71 (m, 4H), 1.48–1.46 (m, *J* = 14.4, 7.1 Hz, 4H), 1.33–1.21 (m, 8H), 0.88 (t, *J* = 6.8 Hz, 3H), 0.68–0.60 (m, 2H). ^13^C NMR (101 MHz, CDCl_3_) δ: 77.36(CH_2_), 62.73(CH_2_), 50.91(CH_3_), 50.66(CH_3_), 50.42(CH_3_), 45.33(CH_2_), 32.01(CH_2_), 29.64(CH_2_), 29.60(CH_2_), 29.42(CH_2_), 29.03(CH_2_), 27.03(CH_2_), 22.80(CH_2_), 20.79(CH_2_), 14.23(CH_3_), 6.83 (CH_2_).

#### 2.1.2 Synthesis of C22

In a flamed flask, 1-chlorodocosane (2.5 g; 7.10 mmol) was dissolved in 32 mL of anhydrous *N*,*N*-dimethylformamide (DMF) under an inert atmosphere. (*N*,*N*-Dimethylaminopropyl)trimethoxysilane (1.6 mL, 7.10 mmol) was added, and then the mixture was stirred at 120 °C for 48 h. When the reaction was finished, the DMF was evaporated under vacuum, and the residue was precipitated with hexane. The product was obtained as a pale yellow solid with 72.28% yield (2.83 g): ^1^H NMR (400 MHz, CDCl_3_) δ 3.63–3.54 (m, 9H), 3.50–3.27 (m, 12H), 1.85–1.62 (m, 8H), 1.41–1.19 (m, 32H), 0.88 (t, *J* = 6.8 Hz, 3H), 0.74–0.65 (m, 2H). ^13^C NMR (101 MHz, CDCl_3_) δ 65.26 (CH_2_), 63.86 (CH_2_), 51.39 (CH_3_), 50.90 (CH_3_), 50.82 (CH_3_), 50.73(CH_3_), 32.05 (CH_2_), 31.71 (CH_2_), 29.84 (CH_2_), 29.79 (CH_2_), 29.72 (CH_2_), 29.60 (CH_2_), 29.49 (CH_2_), 29.38 (CH_2_), 29.02 (CH_2_), 27.02 (CH_2_), 26.49 (CH_2_), 26.42 (CH_2_), 22.90 (CH_2_), 22.82 (CH_2_), 22.78 (CH_2_), 16.63 (CH_2_), 14.24 (CH_3_), 5.71 (CH_2_). C22 was used in a 50 wt % solution in anhydrous methanol.

### 2.2 Zeolite Synthesis

Several synthesis gels were prepared with the following molar composition: 1 Al_2_O_3_: 60 SiO_2_: 11 TPAOH: 1500 H_2_O. First, aluminum isopropoxide (AIP, 98%, Sigma-Aldrich) was mixed at room temperature with deionized water and tetrapropylammonium hydroxide (TPAOH, 40%, Thermo Scientific) in a round-bottom flask until the complete dissolution of AIP. Then, tetraethyl orthosilicate (TEOS, 98%, Sigma-Aldrich) was added dropwise to the AIP solution under magnetic stirring in an ice bath. The hydrolysis of TEOS was performed under stirring for 40 h at room temperature after the ice bath melting. The alcohols, byproducts of hydrolysis of AIP and TEOS, were removed from the synthesis gel by evaporation at 50 °C and 100 mbar. The synthesis gels were aged to promote the formation of protozeolitic nanounits under reflux and magnetic stirring for 20 h at 90 °C.^29^ These entities are formed by aluminosilicate nanoparticles and possess almost the same Si/Al ratio as the starting synthesis gel, accommodating individual molecules and/or clusters of TPA^+^ molecules at internal and external positions. The properties of the protozeolitic units obtained after the aging process have been assessed in detail in a previous work.^30^

After the aging step, the selected organosilane was added in a 5 mol %, related to the initial Si content of the synthesis gel, at 0 °C under magnetic stirring. The addition of the organosilanes at 0 °C provoked the formation of a white emulsion in just a few seconds, except for the C10 organosilane, which remained as a transparent solution. An additional silanization treatment was performed at 90 °C for the C22 organosilane due to its low solubility at 0 °C, driving to the formation of a yellowish dense gel after 3 h of silanization. An aliquot of each silanized synthesis gel was separated, being first vacuum-dried at 50 °C under 5 mbar and then oven-dried overnight at 100 °C.

The silanized synthesis gels were hydrothermally crystallized for 7 days under autogenous pressure into Teflon-lined autoclave reactors at 150 °C. Two well-defined solid phases were found after the hydrothermal crystallization step when using C14 and C18 organosilanes. The upper phases were composed by jellified supernatants,^23^ whereas the lower ones were formed by crystallized ZSM-5 samples. The characterization shown in this work is focused on the lower phase, which was obtained with yields of 63 and 47 wt %, referred to the initial silica-alumina content, when employing the C14 and C18 organosilanes, respectively.

Both parent gels and zeolite samples were calcined using a two-step process.^31^ A first heating step under nitrogen flow (100 mL·min^–1^) was performed at 1.8 °C·min^–1^ up to 400 °C; this temperature was kept constant for 4 h. Then, a second calcination step was carried out at 1.8 °C·min^–1^ up to 550 °C under air flow (100 mL·min^–1^), maintaining this temperature for 5 h.

Zeolitic samples were named referring to the organosilane employed as “ZSM-5 (C*N*)”, where C*N* corresponds to C10, C14, C18, or C22 organosilanes. Samples recovered from the synthesis gel were designated following the same premise as “GEL (C*N*)”. Moreover, as two different silanization temperatures were used with the C22 organosilane, the corresponding gel and zeolite samples were denoted as GEL (C22–0)/ZSM-5 (C22–0) and GEL (C22–90)/ZSM-5 (C22–90), for the materials obtained at silanization temperatures of 0 °C at 90 °C, respectively.

An additional commercial nanocrystalline ZSM-5 zeolite, n-ZSM-5 (Si/Al = 42, Clariant, ref: HCZP90), was employed for comparison purposes.

### 2.3 Materials Characterization

Low- and wide-angle XRD diffraction patterns were collected with an Empyrean PANalytical diffractometer using Cu (Kα = 1.54 Å) covering 2θ ranges of 0°–5° and 5°–50° for low- and high-angle XRD measurements, respectively.

Argon (−186 °C) adsorption–desorption isotherms of calcined samples were measured with a Micromeritics 3Flex instrument. The materials were outgassed under a vacuum at 300 °C for 5 h before the analysis. The specific surface area of the samples was calculated by the BET equation, and the pore size distribution (PSD) was determined by applying the Non-Local DFT (NL-DFT) model to the adsorption branch, assuming cylindrical pores. The total pore volume (*V*_T_) of the samples was estimated at the final relative pressure (*P*/*P*_0_ = 0.99), whereas the micropore volume (*V*_mic_) of each sample was extracted from the NL-DFT cumulative pore volume versus pore size data. The micropore surface area (*S*_mic_) of the zeolitic samples was calculated using a procedure described elsewhere.^32^ The non-microporous surface area (*S*_mes+ext_) and volume (*V*_SP_) were calculated as the difference between the *S*_BET_ and the *S*_mic_, and the *V*_T_ and *V*_mic_, respectively.

ICP-OES analyses were performed to quantify the Si/Al ratio of the calcined zeolites in PerkinElmer Optima 7300 DV equipment. Transmission electron microscopy (TEM) micrographs of the calcined zeolite samples were collected with both JEOL JEM 2100 (200 kV) and JEOL JEM 1400 (120 kV) microscopes; and using a JEOL F200 CF (200 kV) microscope for the gel samples. Scanning electron microscopy (SEM) images of the calcined samples were recorded with a FESEM JEOL microscope operating at 1 kV with GSBH mode.

Solid-state ^29^Si MAS NMR measurements of the as-synthesized samples and ^27^Al MAS NMR analyses of the calcined samples were performed at 79.41 MHz for the former and at 104.26 MHz for the latter, in a Bruker Avance III/HD 400 MHz spectrometer. The concentration of Brønsted and Lewis acid sites (BAS and LAS, respectively) was determined by using pyridine as a probe molecule and monitored by FTIR in a house-made system. Self-supported wafers (15 mg·cm^–2^) were prepared and activated under a vacuum (10^–4^ mbar) at 525 °C for 4 h prior to the measurements. Thereafter, pyridine was introduced into the system at 150 °C, which was kept closed for 20 min. Thermal desorption was performed under a high vacuum at increasing temperatures in the range 150–450 °C (heating rate: 10 °C·min^–1^), applying a 20 min equilibrium period before taking the spectrum at each selected temperature. Spectra were recorded using a Jasco-4600 instrument equipped with a TGS detector, with a resolution of 4 cm^–1^ and 128 scans. The integrated molar extinction coefficients used for the BAS and LAS concentration quantification were taken from Zholobenko et al.^33^ for the ZSM-5 zeolite: ξ_BAS_ = 1.09 cm·μmol^–1^ and ξ_LAS_ = 1.71 cm·μmol^–1^.

### 2.4 Catalytic Performance Evaluation

Catalytic tests of furfural (FFL) and cyclopentanone (CPO) aldol condensation were carried out in a StartFish reaction system (5 × 25 mL flasks) at 80 °C. Every single flask was loaded with 10.1 and 1.15 g of CPO (Sigma-Aldrich, 99%) and FFL (Sigma-Aldrich, 99%), respectively (CPO:FFL molar ratio: 10:1) and maintained under vigorous stirring (750 rpm). When the reaction temperature was reached, 0.2 g of the zeolite sample was added, assuming that FFL and CPO evaporation is negligible since the reaction temperature is much lower than the boiling point of these compounds. However, once the catalyst was introduced, the condensation system was connected to avoid any volatilization from the reaction medium. 0.2 mL was withdrawn from the reaction medium at different times (2, 4, and 6 h), filtered, and diluted (1:10 mass ratio) in a mixture of ethyl acetate (solvent) and 2000 ppm of mesitylene (internal standard) to be quantitatively analyzed in an Agilent 7890A GC equipped with FID detector and a HP-5 30 m column. The identification of the main products was confirmed using the GC-MS (Agilent 8860 GC-5977B) technique with HP-5MS UI column.

**Scheme 1:**
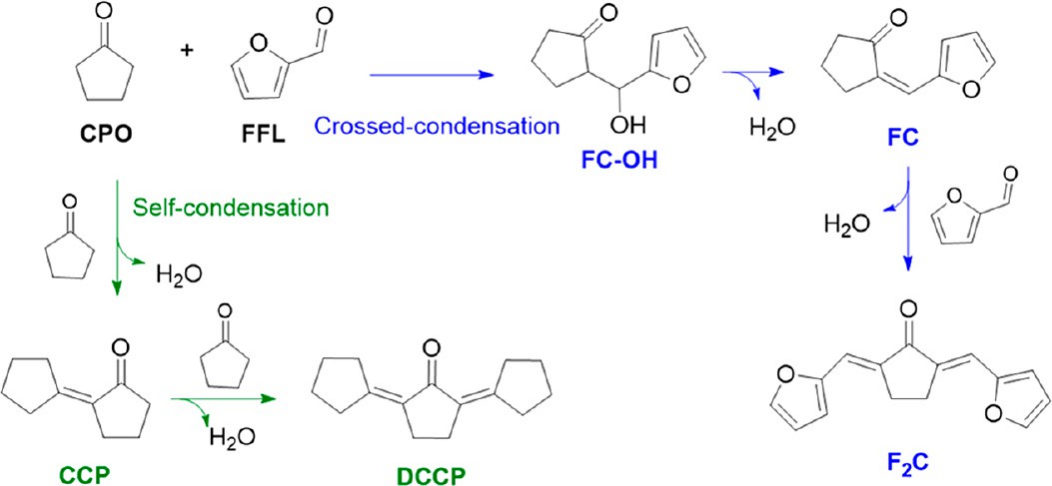
Main Products Obtained in the Crossed-Condensation of Furfural and Cyclopentanone (blue route) and Self-Condensation of Cyclopentanone (green route) Catalyzed by Zeolites

Condensation adducts (FC and F_2_C, see Scheme 1) are not commercially available, and preparation is based on previous works.^34^ A mixture of FFL (3 g, 31.23 mmol), CPO (3.15 g, 37.47 mmol), diethyl ether (31 mL), and a 1 M NaOH solution (31 mL) was stirred at room temperature for 24 h. The mixture was diluted with 50 mL of ethyl acetate (EtOAc). The aqueous layer was separated and extracted with EtOAc (3 × 20 mL). Then, the aqueous phase was filtered under vacuum to obtain F_2_C (680.8 mg) product as a yellow solid: ^1^H NMR (400 MHz, CDCl_3_) δ 7.60 (d, *J* = 1.6 Hz, 2H), 7.36 (s, 2H), 6.71 (d, *J* = 3.4 Hz, 2H), 6.55 (dd, *J* = 3.4, 1.6 Hz, 2H), 3.10 (s, 4H). The organic phase was washed with water (3 × 20 mL) and dried over Mg_2_SO_4_. The solvent was evaporated, and the residue was purified by silica gel column chromatography with hexane: EtOAc (10:1) to obtain 2.257 g (44.57% yield) of FC adduct as a yellow solid and 828.7 mg of F_2_C (6.28 mmol, 40.21% yield): ^1^H NMR (400 MHz, CDCl_3_) δ 7.57 (d, *J* = 1.8 Hz, 1H), 7.18 (t, *J* = 2.7 Hz, 1H), 6.67 (d, *J* = 3.4 Hz, 1H), 6.52 (dd, *J* = 3.4, 1.8 Hz, 1H), 3.00 (td, *J* = 7.3, 2.6 Hz, 2H), 2.41 (t, *J* = 7.6 Hz, 2H), 2.04 (q, 7.6 Hz, 2H). The purity of these compounds was checked by ^1^H NMR analyses (Figure S1), which are used as analytical standards in the GC calibration method. When the reaction was finished, the liquid reaction mixture was filtered and the catalyst was recovered and dried overnight. Once the concentrations of the main compounds involved in this reaction were obtained, results were expressed in terms of conversion, turnover frequency (TOF), and product selectivity (*S*_*i*_) calculated following eqs 1–3:
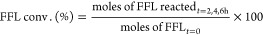
1
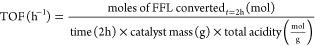
2
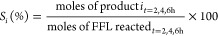
3

## 3 Results and Discussion

### 3.1 Characterization of Precursor Gels

**Figure 1:**
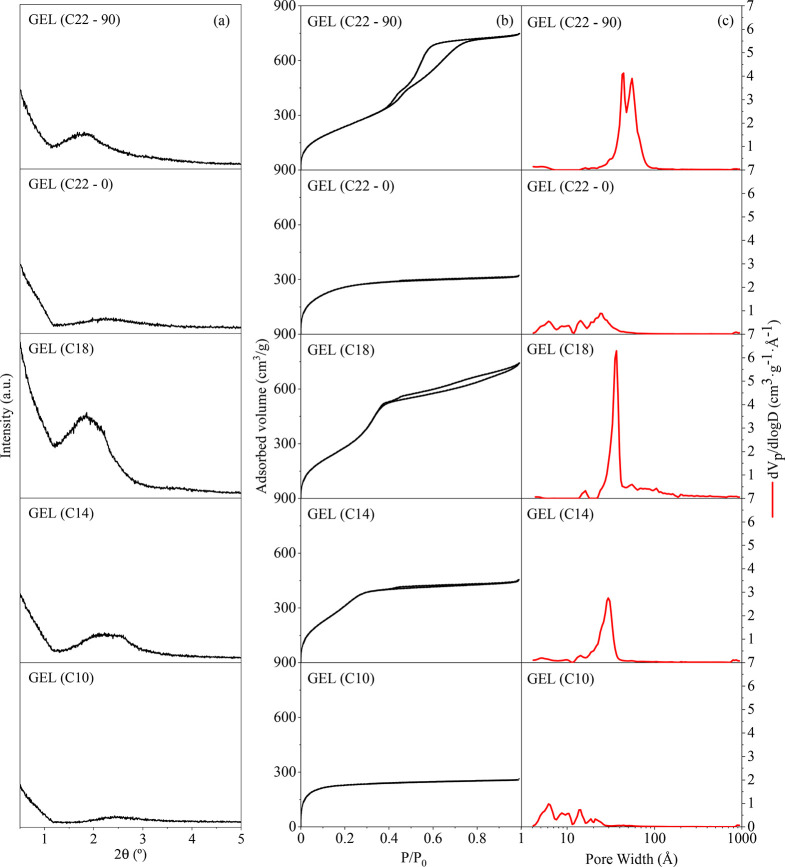
As-prepared gel samples: low-angle XRD patterns (a). Calcined gel samples: Ar adsorption–desorption isotherms at −186 °C (b) and NL-DFT PSD (c).

Samples recovered from the silanized synthesis gels were first characterized by low-angle XRD (Figure 1a) to probe the possible presence of mesoscale ordering before the hydrothermal crystallization stage. Broad diffraction peaks can be appreciated in the low-angle XRD patterns of both GEL (C14) and GEL (C18), which can be considered as indicative of the formation of ordered mesophases in the early stages of the synthesis. On the contrary, low-angle XRD reflections are practically absent in the case of GEL (C10) and GEL (C22–0) samples. These results suggest that the appearance of mesophases may strongly depends on the length of the organosilane alkyl chain, which can be related with its ability to form micelles. Thus, for samples GEL (C14) and GEL (C18), the surfactant properties of the amphiphilic organosilane are suitable for the formation of micelles, acting as templates for the mesophase generation. However, an insufficient alkyl chain length (i.e., C10 organosilane) may hamper the formation of the mesophase due to its lower hydrophobicity, which increases the critical micelle concentration. On the other hand, a too long alkyl chain, as for the GEL (C22–0) sample, may lead to a reduced solubility of the surfactant in the aqueous medium of the synthesis gel, thus hindering its contact with the protozeolitic nanounits. Accordingly, and with the aim of increasing the solubility of the C22 organosilane in the synthesis medium, a second gel was prepared by silanization at higher temperature (90 °C). The low-angle XRD patterns of GEL (C22–90) sample shows a broad but clear diffraction signal, denoting that the increase of the silanization temperature has been effective for the mesophase formation. Nevertheless, the low angle X-ray reflections are not sufficient for pointing out conclusively the presence of an ordered mesophase after silanization. Thus, argon (−186 °C) adsorption–desorption isotherms were collected in order to obtain the PSD of the samples.

Textural properties of the gel calcined samples were measured by the Ar adsorption–desorption experiments (−186 °C), applying the NL-DFT model to obtain the corresponding PSD (Figure 1b,c, respectively). Both GEL (C10) and GEL (C22–0) samples display type I(b) isotherms, characteristic of solids having mostly micropores.^35^ Moreover, they exhibit PSD that resembles those of ZSM-5 zeolite synthesis gels obtained when employing non-surfactant organosilanes,^30^ with several peaks being observed in the micropore range and at the micro/mesopore border. These results are in good agreement with the absence of any ordered mesophase in GEL (C10) and GEL (C22–0), as above suggested by the low-angle XRD patterns. In contrast, GEL (C14) and GEL (C18) show well-defined type IV isotherms characteristic of ordered mesoporous materials. In this way, as can be appreciated in the PSD curves (Figure 1c), both samples possess narrow mesopores with peak maximum at about 30 and 37 Å for GEL (C14) and GEL (C18), respectively. Therefore, a direct relationship seems to exist between the mesopore size of the so-formed mesophases and the alkyl chain length of the organosilane. The presence of uniform mesoporosity can be clearly observed also in the TEM micrographs of GEL (C14) and GEL (C18) (Figure S2), exhibiting distinctive wormhole type mesopores that are homogeneously distributed throughout the gel samples. Regarding GEL (C22–90), the PSD confirms the mesophase formation with the longest chain organosilane when using silanization temperatures high enough for improving its dispersion within the gel. The PSD of GEL (C22–90) shows a bimodal mesopore size distribution, with maximum at ca. 44 and 55 Å, evidencing the occurrence of a complex synthesis system probably associated with the gelification of the raw mixture that occurred after 3 h of silanization at 90 °C.

As shown in Table 1, all the gel samples possess a high BET surface area with values over 700 m^2^ g^–1^, the largest ones corresponding to GEL (C14) and GEL (C18) (1028 and 953 m^2^ g^–1^, respectively). More pronounced differences are observed between the gels in terms of total pore volumes, varying in the ranges of 0.336 cm^3^ g^–1^ (GEL (C10)) and 0.953 cm^3^ g^–1^ (GEL (C22–90)). Interestingly, this last sample presents a total pore volume very superior to that of GEL (C22–0) (0.412 cm^3^·g^–1^), which can be associated with the mesophase formation.

**Table 1:** Textural Properties of Calcined Gel Samples Derived from the Ar Adsorption–Desorption Isotherms (−186 °C)

Sample	S_BET_^a^ (m^2^·g^–1^)	*V*_T_^b^ (cm^3^·g^–1^)	*V*_mic_^b^ (cm^3^·g^–1^)	*V*_mes_^b^ (cm^3^·g^–1^)
GEL (C22–90)	794	0.953	0.04	0.913
GEL (C22–0)	861	0.412	0.31	0.102
GEL (C18)	953	0.947	0.04	0.907
GEL (C14)	1028	0.580	0.15	0.43
GEL (C10)	736	0.336	0.31	0.025

a *S*_BET_: BET surface area.

b *V*_T_, *V*_mic_, and *V*_mes_: total, micropore (<20 Å), and mesopore volumes, respectively.

The existence of a mesophase with relatively uniform mesopores, centered at larger diameters as the organosilane alkyl chain length increases, denotes the formation of micelles during the silanization treatment. It can be envisaged that the protozeolitic nanounits are assembled around these micelles, forming a Pickering-like emulsion^36^ stabilized by the occurrence of covalent bonding. This assumption is confirmed by the presence of T species in the ^29^Si MAS NMR spectra (Figure S3) of the as-synthesized gels when C10, C14, and C18 organosilanes are employed, arising from the grafting of the organosilane to the aluminosilicate nanounits. These T signals appearing between the chemical shifts of −48 and −65 ppm in the ^29^Si MAS NMR spectra reveal Si(OSi)(OR)_2_, R′Si(OSi)_2_(OR), and R′Si(OSi)_3_ silicon moieties, typically called T^1^, T^2^ and T^3^ species, respectively. Also, the second group of signals in the ^29^Si MAS NMR measurements between chemical shifts of −85 and −110 ppm are attributed to Q^1^ [Si(O^–^)(OH)_3_], Q^2^ [Si(O^–^)_2_(OH)_2_], Q^3^ [Si(O^–^)_3_(OH)], and Q^4^ [Si(O^–^)_4_] silicon species of the aluminosilicate zeolitic structure. Although the distribution of Q species does not follow any particular tendency between the gel samples, the total molar concentration of covalently bonded organosilane (T species) increases as the organosilane alkyl chain is enlarged for C10, C14, and C18, showing a higher degree of functionalization of the zeolite embryos. On the other hand, the almost negligible signal of T species in the ^29^Si MAS NMR spectra of GEL (C22–0) sample (Figure S3) indicates that, at least during the silanization stage, this organosilane is little anchored onto the protozeolitic units, probably due to its high hydrophobicity. GEL (C22–90) displays a more intense, but still low, signal of T species, showing that increasing the silanization temperature, although it improves the dispersion of the C22 organosilane and affords the formation of mesophases, is not sufficient to establish a noticeable proportion of covalent bonds with the protozeolitic nanounits.

### 3.2 Properties of the ZSM-5 Samples

**Figure 2:**
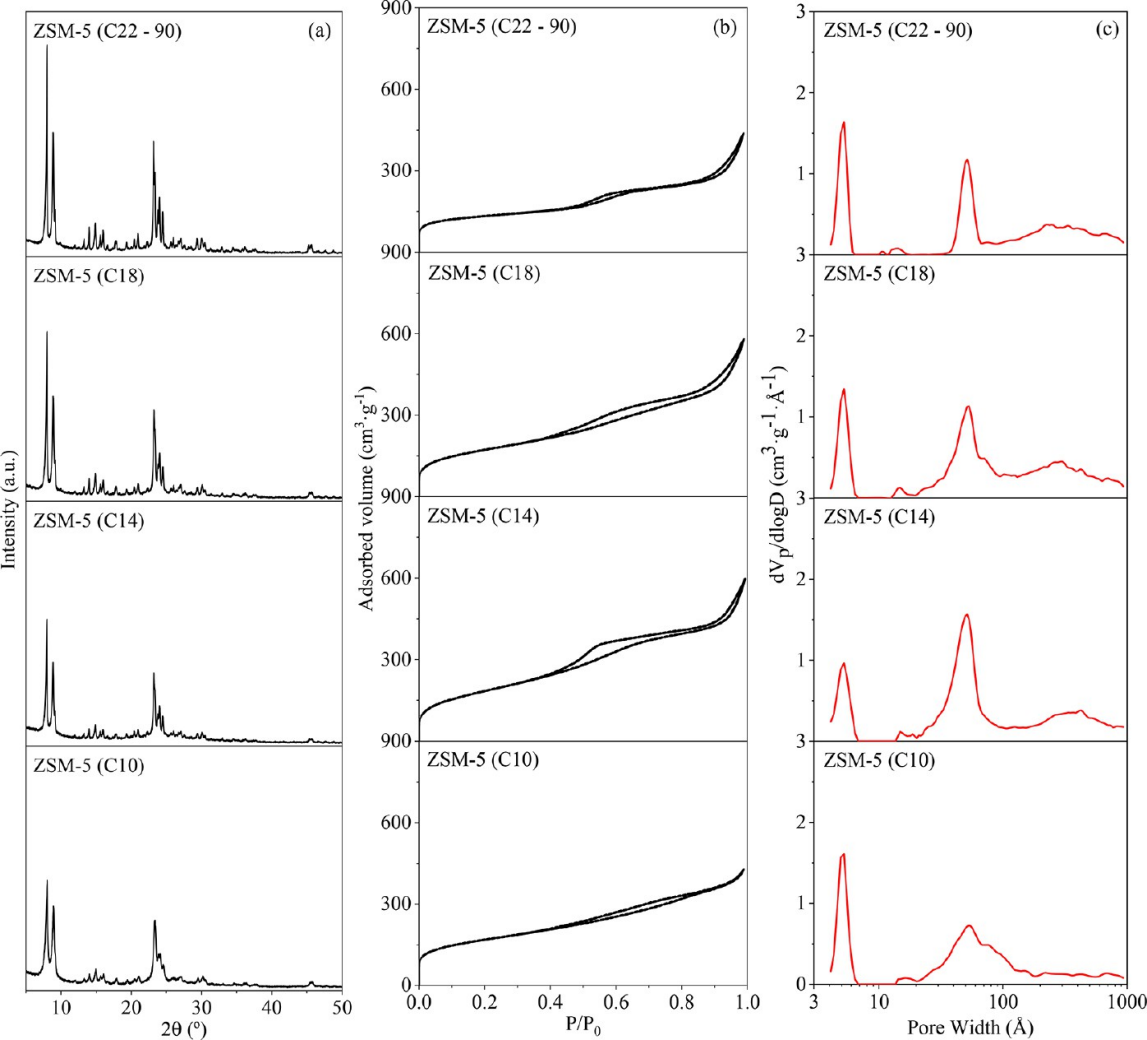
XRD patterns (a), argon isotherms (−186 °C) (b), and NL-DFT PSDs (c) of the calcined ZSM-5 samples.

After the silanization step, hydrothermal crystallization of the previously obtained synthesis gels was performed at 150 °C for 7 days. The XRD pattern of the ZSM-5 (C22–0) sample (Figure S4a) shows, in addition to peaks characteristic of the MFI structure, the presence of an amorphous halo, indicating an incomplete crystallization. This fact is also confirmed by the TEM micrographs, in which the coexistence of amorphous (Figure S4b) and crystalline (Figure S4c) phases is observed, the latter consisting of a loose packing of small ZSM-5 nanocrystals. In contrast, for the rest of the samples, the amorphous halo is absent in the XRD patterns (Figure 2a, showing that they are highly crystalline zeolites).Interestingly, the intensity and definition of the XRD peaks are enhanced as the organosilane chain length increases, indicating that the size of the crystalline domains is progressively enlarged from C10 to C22 samples. This finding can be directly related with the decrease of the organosilane hydrophobicity when shortening the chain length, as it may favor its contact with the protozeolitic nanounits, preventing in great part their aggregation into larger crystalline particles. In addition, the Scherrer equation^37^ has been applied to the most intense X-ray diffractions (2θ at 8°, 8.9°, 23.4°, 24.1°, and 24.5°) for obtaining information about the sizes of the crystalline domains within the zeolitic particles. The average values so obtained were 24, 45, 40, and 58 nm for the ZSM-5 (C10), ZSM-5 (C14), ZSM-5 (C18), and ZSM-5 (C22–90) samples, respectively. Accordingly, a trend toward larger crystalline domains can be appreciated when increasing the length of the organosilane. Interestingly, the sizes of the crystalline domains for the samples prepared with C14 and C18 organosilanes are clearly larger than that of the nanounits observed in the TEM images (about 10 nm, as commented on below), showing the occurrence of a high degree of aggregation of the latter. Nevertheless, it has to be noted that the Scherrer equation can be sharply influenced by crystal lattice imperfections and/or deformations, as it will likely occur when grafting the organosilane molecules to the protozeolitic entities; hence, the obtained values should be interpreted rather in a semiquantitative way.

**Figure 3:**
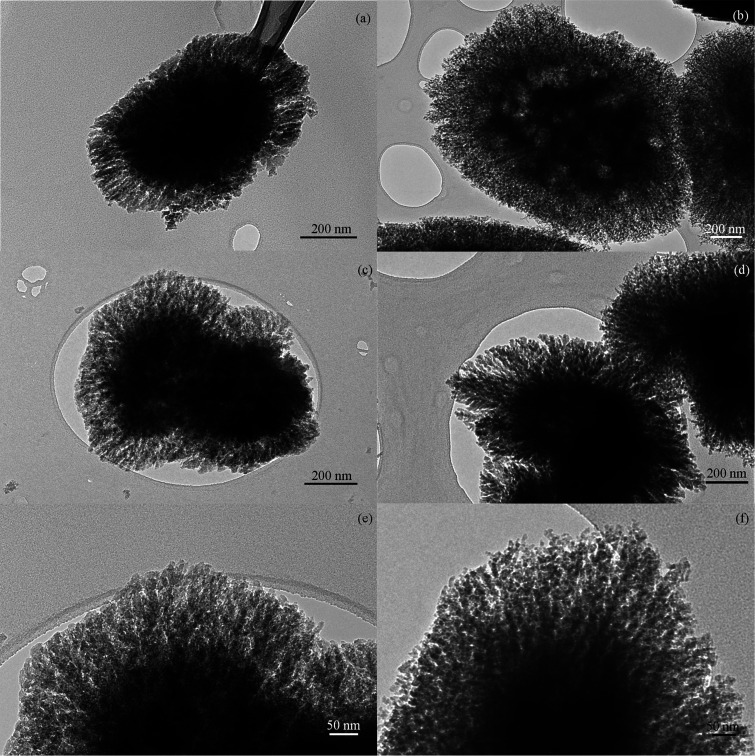
TEM micrographs of ZSM-5 (C14) (a, c, e) and ZSM-5 (C18) (b, d, f) samples.

Remarkably, ZSM-5 materials featuring dendritic nanoarchitectures are produced when using C14 and C18 organosilanes, as shown by the TEM images (Figure 3). These samples are formed by particles with sizes in the range 400–1000 nm, consisting of aggregates of ca. 10 nm nanounits arranged in a radially oriented pattern with a tree-like branching. Moreover, the presence of relatively large cavities can be observed inside the dendritic aggregates. On the contrary, ZSM-5 (C10) sample consists of globular particles (Figure S5), formed by randomly aggregated nanocrystals in which the radial orientation is mostly missing. In fact, this sample shows the typical morphology of a hierarchical ZSM-5 zeolite synthesized using non-surfactant organosilanes during the functionalization of the protozeolitic nanounits.^29,38−40^ Finally, relatively larger nanocrystal aggregates, with sizes up to the micrometer range, can be observed for the ZSM-5 (C22–90) sample (Figure S6). The shape of these particles tends to be rectangular, resembling the typical coffin morphology of micrometric crystals in conventional ZSM-5, but without any evidence of dendritic architecture.

**Figure 4:**
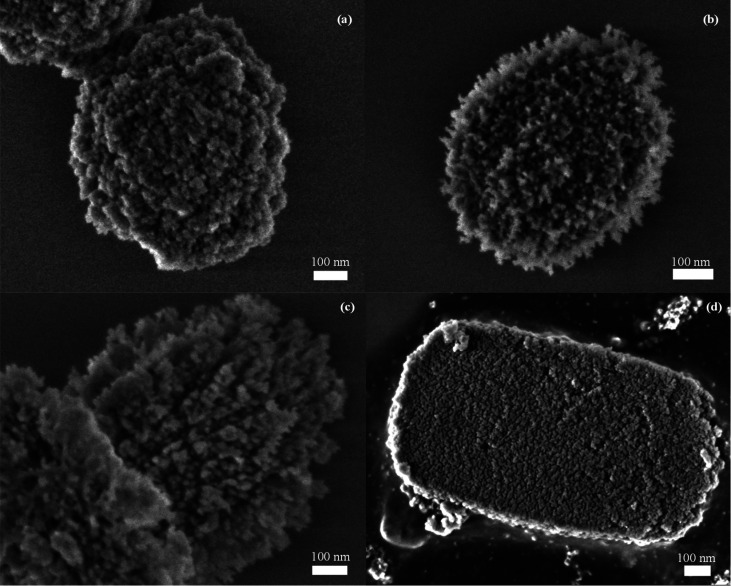
SEM micrographs of ZSM-5 (C10) (a), ZSM-5 (C14) (b), ZSM-5 (C18) (c), and ZSM-5 (C22–90) (d) samples.

Additional insights into the morphological differences between the zeolite samples can be observed in the SEM images (Figure 4). The surface of the particles for ZSM-5 (C10) sample is relatively compact, showing some rugosity due to the presence of smaller units randomly aggregated. In contrast, for ZSM-5 (C14) and ZSM-5 (C18) zeolites, the surface of the particles exhibits a high porosity and roughness as a consequence of the dendritic nanoarchitecture. Finally, the ZSM-5 (C22–90) sample displays a quite flat surface, but with distinctive intricated porosity (Figure 4d), differing considerably from the smooth surface of a conventional ZSM-5 zeolite. As previously mentioned, during the C22 silanization stage at 90 °C, the gelation of the synthesis mixture occurred. This drastic change of the synthesis gel creates an in situ hard-templating environment, hence the zeolite crystallization proceeds in a confined space.

The porous structure and textural properties of the samples can be extracted from the Ar adsorption–desorption isotherms and the corresponding PSD (Figure 2b,c, respectively). ZSM-5 (C10) exhibits a hybrid type I–IV isotherm with bimodal PSD, consisting of zeolitic micropores and a broad mesoporosity (from 2 to 10 nm), as it has been frequently observed in hierarchical ZSM-5 zeolites prepared using non-surfactant organosilanes.^30^ For the other three zeolite samples, the isotherms are more complex, showing three main adsorption zones at low, intermediate, and high relative pressures. In this way, a trimodal PSD is obtained for these materials by applying the NL-DFT model to the adsorption branch of the isotherm: zeolitic micropores (at ca. 0.55 nm), narrow mesopores (centered at about 5 nm), broad mesopores (20–50 nm), and macropores extending from 50 nm. The presence of highly uniform mesopores at 5 nm can be linked to the formation of mesophases in the gels corresponding to the samples prepared with C14, C18 and C22 organosilanes. The contribution of this mesoporosity is especially relevant for the ZSM-5 (C14) and ZSM-5 (C18) samples; hence, in this case it can be related to the voids existing within the dendritic array of nanounits. Moreover, the presence of a narrow mesoporosity is an indication that the zeolites are crystallized directly from the mesophases existing in the gels. Nevertheless, the mesopores in the ZSM-5 materials exhibit higher dimensions with respect to the parent synthesis gel, showing that a strong distortion of the micelles in the mesophases occurs during the zeolite crystallization process, which is consistent with the absence of any low-angle XRD peak in the final ZSM-5 samples (data not shown). On the other hand, the porosity present in the border between meso- and macropores in the PSD can be linked to the large vesicles/cavities observed in the TEM images, probably generated by the presence of micelle aggregates in the synthesis gel, as well as to interstitial voids.

**Figure 5:**
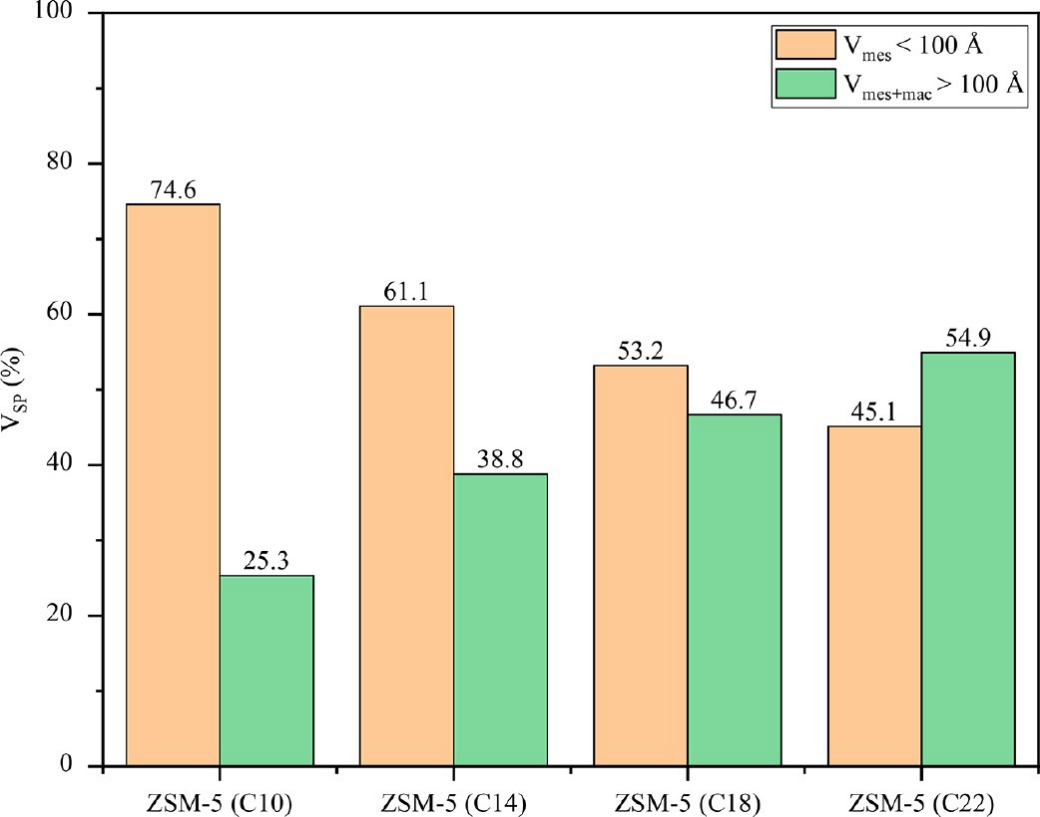
Pore volume share of mesopores smaller than 100 Å (*V*_mes_) and meso/macropores larger than 100 Å (*V*_mes+mac_).

Regarding the textural properties (Table 2), all zeolite samples possess a significant contribution of the porosities corresponding to meso- and macropores, as denoted by their high external/mesopore surface. Interestingly, this effect is more pronounced for the zeolite materials showing a dendritic nanoarchitecture, which also exhibit enhanced BET surface area and overall pore volumes. In this way, high BET surface areas (590 and 550 m^2^·g^–1^) are observed for the ZSM-5 (C14) and ZSM-5 (C18) samples, with a share of the external/mesopore surface area of 67% and 59%, respectively. On the other hand, a direct relationship has been found between the organosilane alkyl length and the contribution, in terms of pore volume, of the two types of secondary porosities present in the samples. As illustrated in Figure 5, the share of the mesoporosity centered at about 5 nm in the PSD tends to decline as the organosilane chain length is increased, passing from representing 75% of the overall secondary porosity for ZSM-5 (C10) to 45% for ZSM-5 (C22–90). This variation can be attributed to a progressive enhanced presence of micellar aggregates due to the higher hydrophobic character of the organosilane as the alkyl chain length is larger.

**Table 2:** Textural Properties of Calcined Zeolite Samples Derived from Ar Adsorption–Desorption Isotherms (−186 °C)

Sample	*S*_BET_^a^ (m^2^·g^–1^)	*S*_mic_^a^ (m^2^·g^–1^)	*S*_mes+ext_^a^ (m^2^·g^–1^)	*V*_T_^b^ (cm^3^·g^–1^)	*V*_mic_^b^ (cm^3^·g^–1^)	*V*_SP_^b^ (cm^3^·g^–1^)
ZSM-5 (C22–90)	414	241	173	0.558	0.150	0.408
ZSM-5 (C18)	550	225	325	0.739	0.140	0.599
ZSM-5 (C14)	590	193	397	0.761	0.120	0.641
ZSM-5 (C10)	532	265	267	0.544	0.165	0.379

a *S*_BET_, *S*_mic_, and *S*_mes+ext_: BET, micropore, and mesopore/external surface areas, respectively.

b *V*_T_, *V*_mic_, *V*_SP_: total, micropore volume (<7.5 Å), and secondary porosity volumes, respectively.

^29^Si MAS NMR spectra of the as-synthesized ZSM-5 samples are shown in Figure S7, providing interesting information about the incorporation of the organosilanes onto the zeolite surface. In the particular case of ZSM-5 (C22–90), a very small contribution of T species is observed, evidencing that C22 organosilane is not covalently linked to the zeolite phase during the hydrothermal crystallization, as it also occurred in great part for its precursor gel. As indicated above, with this organosilane the synthesis gel condensed abruptly during the silanization treatment, hindering the contact between the silanization agent and the protozeolitic nanounits. This is not the case for the rest of the samples as they show a noticeable population of T species. A slight reduction of the T-species share, from 4.4 to 3.5%, was found comparing the GEL (C10) sample and the ZSM-5 (C10) zeolite. In contrast, T-species population of the ZSM-5 (C14) and ZSM-5 (C18) samples significantly increases regarding the parent gels. For the sample synthesized with the C14 organosilane, the proportion of the T-species increases from 6.9 in the gel to 12.4% in the final zeolite sample. Similarly, an increment from 7.4 to 9.6% was evidenced when comparing the GEL (C18) and the ZSM-5 (C18) samples, respectively. These results can be explained by a segregation of the silanized synthesis gel when employing the C14 and C18 organosilanes. The apparently homogeneous whitish emulsion, generated by the addition of these organosilanes to the gel, rapidly undergoes phase separation when stirring is stopped, leading to a white gel phase at the bottom zone and a more transparent liquid in the upper part of the flask. In light of the ^29^Si MAS NMR results, it can be concluded that the lower phase contains the major part of the organosilane added to the synthesis gel, along with a part of the protozeolitic units, mainly those covalently anchored during silanization. Other interesting fact, which can be derived from the ^29^Si MAS NMR spectra, is that the dendritic samples present a less condensed zeolitic structure than the ZSM-5 (C10) and ZSM-5 (C22–90) zeolites, as supported by the distribution of Q species, at chemical shifts between −85 and −110 ppm. Thus, the share of Q^4^ species is about 54–56% for ZSM-5 (C14) and ZSM-5 (C18), while it increases up to ca. 66–69% for ZSM-5 (C10) and ZSM-5 (C22–90). In terms of Q^4^/Q^3^ ratio, this implies a variation from 1.85 for the dendritic ZSM-5 samples to about 3.3 for the other two materials. This important difference can be assigned to the high degree of grafting achieved with the C14 and C18 organosilanes, which largely prevents the condensation of the zeolitic entities.

Concerning the aluminum content and acid properties of the calcined zeolites, Table 3 summarizes their Si/Al ratio and the concentration of Brønsted (*C*_B_), Lewis (*C*_L_) and total acid sites (*C*_T_) as well as the Brønsted/Lewis sites concentration ratio (*C*_B_/*C*_L_). A different aluminum incorporation (Si/Al ratios from 31.5 to 42.7) was observed for the different samples, with lower Al contents being present in the dendritic ZSM-5 samples. Likewise, important changes are denoted among the samples according to the *C*_B_/*C*_L_ ratio, which varies from 0.76 for the ZSM-5 (C18) zeolite to 1.81 for the ZSM-5 (C22–90) sample. The fact that the dendritic zeolites exhibit lower *C*_B_/*C*_L_ ratios can be linked with its high share of mesopore/external surface area, being in agreement with earlier results connecting both parameters.^29,41^ This Lewis acidity seems to be originated mainly by framework Al species, since just a small content of Al atoms with octahedral coordination (signal at 0 ppm) has been detected in the ^27^Al MAS NMR spectra of the calcined samples (Figure S8). Thus, the share of octahedral Al species remains in the range 2.4–5% for the different materials, indicating that most of the Al atoms are incorporated into the zeolite framework.

**Table 3:** Si/Al Ratio (from ICP-OES measurements) and Brønsted (*C*_B_) and Lewis (C_L_) Acid Site Concentrations (from FTIR-pyridine tests at 150 °C) of the Zeolite Samples

Sample	Si/Al	*C*_B_ (mmol·g^–1^)	*C*_L_ (mmol·g^–1^)	*C*_B_/*C*_L_
ZSM-5 (C22–90)	31.5	0.179	0.099	1.81
ZSM-5 (C18)	42.7	0.122	0.161	0.76
ZSM-5 (C14)	36.6	0.164	0.147	1.11
ZSM-5 (C10)	32	0.248	0.169	1.47

### 3.3 Catalytic Performance in the Furfural-Cyclopentanone Aldol Condensation

Furfural (FFL) and cyclopentanone (CPO) are platform molecules derived from biomass and can be used as reactants in aldol condensation transformations, producing two main adducts that contain 10 and 15 carbon atoms in their structure, respectively.^42^ Taking into account the bulky nature of these species, it can be anticipated that strong diffusional and steric limitations may occur when purely microporous zeolites are used as catalysts. Therefore, this reaction is both an industrially interesting process and a suitable test for zeolite samples with enhanced accessibility.

**Figure 6:**
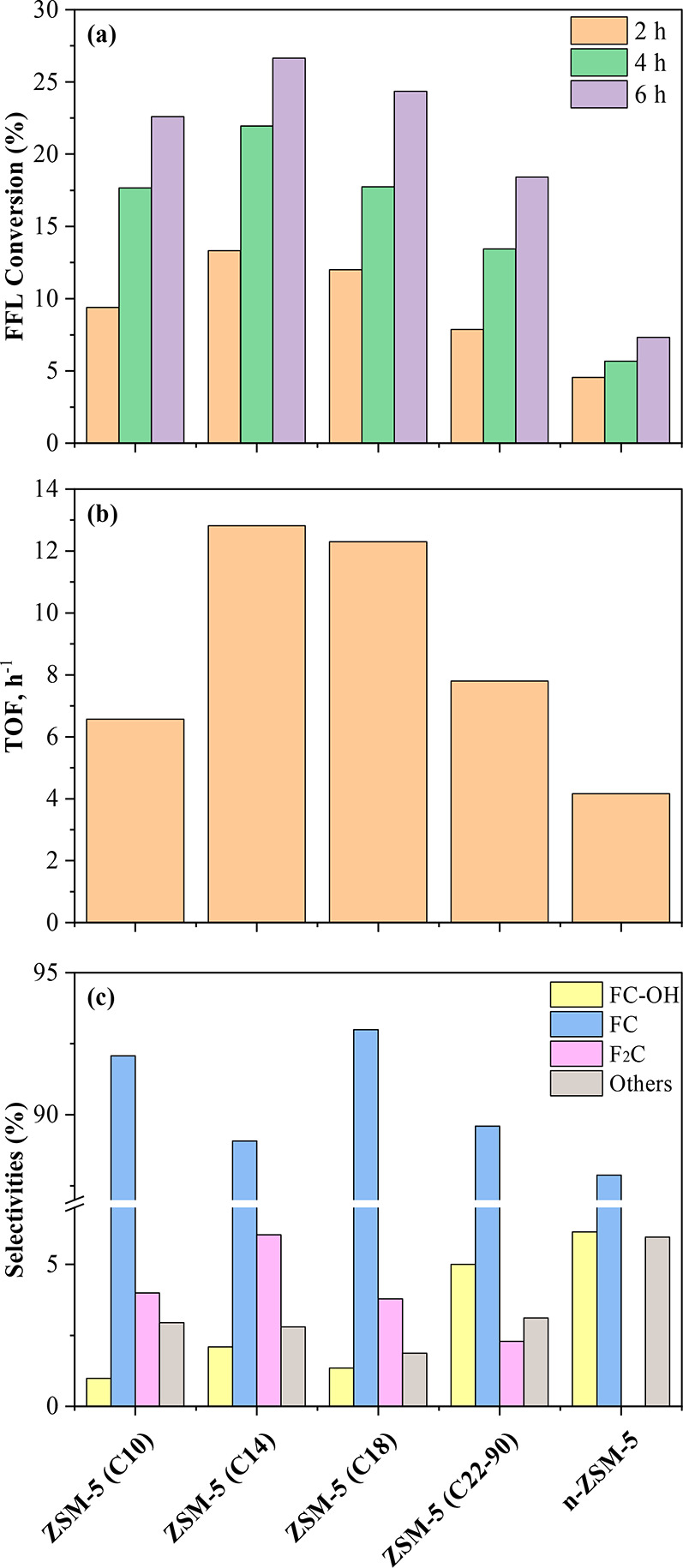
CPO and FFL aldol condensation: (a) evolution of FFL conversion with the reaction time, (b) TOF calculated at 2 h of reaction time, and (c) products selectivity at 6 h of reaction time.

Figure 6 shows the results obtained in FFL and CPO aldol condensation over the ZSM-5 samples prepared using organosilanes with different chain length. In addition, a commercial nanocrystalline sample (n-ZSM-5) was used as a reference (properties shown in Table S1). Since CPO is present in high excess, playing the role of reactant and solvent at the same time, the results are interpreted based on FFL conversion. As expected, this parameter increases progressively with the reaction time, indicating significant differences between the tested materials (Figure 6a). The highest FFL conversions (26.7% at 6 h) are achieved with ZSM-5 (C14) and gradually descends in the following order: ZSM-5 (C18) > ZSM-5 (C10) > ZSM-5 (C22–90) ≫ n-ZSM- 5. This last material presents a low catalytic activity in this reaction, with just a slow increase in FFL conversion with time (from 4.6 to 7.3% at 2 and 6 h, respectively). These variations are even more accentuated in terms of TOF values, calculated from FFL conversion values at 2 h of reaction time and referred to the overall zeolite acidity (Figure 6b). This graph confirms the quite superior catalytic activity of the dendritic zeolites (ZSM- 5 (C14) and ZSM-5 (C18) samples). This result can be assigned first to the remarkable accessibility of these materials derived from their high mesopore/external surface area and trimodal porosity that allows a more facile interaction between the reactants and the active centers. Nevertheless, the acid properties of the zeolite samples may also influence the catalytic results considering all the transformations that can take place during the aldol condensation tests. On one hand, FFL polymerization and dehydration reactions are expected to be catalyzed by Brønsted acid sites.^43,44^ On the other hand, the FFL addition steps are promoted by the presence of Lewis acid sites.^45,46^ Thus, both types of acid centers may participate in the aldol-condensation reaction scheme, which could also explain the good performance observed with the dendritic ZSM-5 zeolites, as they present a proper balance of Brønsted and Lewis acidities.

Regarding the product distribution, four main compounds are observed by GC-MS analysis: FC–OH, FC, CCP and F_2_C (Scheme 1). These species can be obtained from two different reaction pathways: FFL and CPO cross-aldol condensation and CPO self-condensation. Both routes occur through a keto–enol tautomerization process of ketone to form enol species. Then, the enol can react with other molecules of CPO or FFL, producing β-hydroxy acetones (hydrated molecules). Subsequently, dehydration proceeds to obtain the α,β-unsaturated products (molecules with 10 carbon atoms).^47,48^ Under the tested reaction conditions and using the ZSM-5 zeolite catalysts, the crossed-condensation reaction rate is much faster, leading to the formation of FC and even of the second condensation adduct (F_2_C) when another FFL molecule interacts with FC. On the contrary, DCCP moieties have not been detected, whereas the CCP is observed just in small amounts for all the samples tested. These results are in agreement with earlier literature since higher temperatures are necessary to promote self-aldol reaction.^49,50^ Regarding the crossed-aldol reaction, FC is the most abundant product with selectivity above 85% in all cases. In particular, ZSM-5 (C18) and ZSM-5 (C10) exhibit the highest FC selectivity (>90%). For ZSM-5 (C14), the F_2_C selectivity is maximized (6%), confirming that this material is the most active under these reaction conditions. In contrast, FC-OH formation is favored over ZSM-5 (C22–90) and n-ZSM-5 samples (5 and 6.1%, respectively). In this way, F_2_C is observed over ZSM-5 (C22–90) with low selectivities (2.3%), whereas it is not detected for the n-ZSM-5 sample. The sum of selectivities to the mentioned products is not 100% since carbonaceous deposits are formed on the catalysts (the color of the spent zeolites turns to brown for ZSM-5 (C10, C14, C18 and C22–90) and black for n-ZSM-5). This fact can be related to the formation of oligomers and other heavy products that remain deposited over the zeolite catalysts. The highest production of these undesired species (6% selectivity) occurs with n-ZSM-5 zeolite, which can be assigned to this material possessing a higher proportion of micropores, in which bulky compounds are retained. In contrast, their formation is significantly lower for the dendritic zeolites (specially over the n-ZSM-5 (C18) sample), which can be also linked to the singular combination of accessibility and balanced Brønsted/Lewis acidity in these materials.

## 4 Conclusions

Dendritic ZSM-5 zeolites, characterized by a branched/radially oriented nanoarchitecture and showing enhanced textural properties and accessibility, can be prepared by functionalization of protozeolitic nanounits with amphiphilic organosilanes with an appropriate chain length. The generation of the dendritic nanoarchitecture can be related to the formation of a mesophase during the gel preparation, linked through covalent interactions to the zeolite embryos, which specifically occurs using both C14 and C18 organosilanes. Further, this type of bond remains after the gel hydrothermal treatment, suggesting that the crystallization of the corresponding dendritic zeolites (ZSM-5 (C14) and ZSM-5 (C18)) is mediated by the covalently stabilized mesophase (micellar soft templating). The micellar aggregation of the organosilanes is therefore an essential precondition to form an emulsion in the synthesis gel that ultimately leads to dendritic nanoarchitectures after the hydrothermal treatment.

When the organosilane chain length is too short, GEL (C10), its reduced surfactant ability is not enough for the formation of any uniform mesophase, although covalent bonds seem to be effectively established between the silanization agent and the protozeolitic nanounits, as demonstrated by ^29^Si MAS NMR measurements. After the hydrothermal crystallization, this gel leads to the formation of a hierarchical zeolite (ZSM-5 (C10)) consisting of the random aggregation of crystalline nanounits, but lacking of clear dendritic features (molecular soft-templating). Thus, the presence of covalent bonding is a necessary condition but not sufficient to form dendritic nanoarchitectures.

In contrast, using an organosilane with a long hydrocarbon tail (C22), the gel obtained at 0 °C does not show any uniform mesophase, and the covalent bonding with the zeolite embryos is hardly observed, which could be explained by its high hydrophobicity. In this case, increasing the silanization temperature from 0 to 90 °C affords the formation of a mesophase but still lacking of covalent bonding, as no quantifiable presence of T species has been detected in the ^29^Si MAS NMR spectra. Moreover, the transformation of this gel into more dense aggregates of crystalline nanounits occurs during the silanization treatment, which means that the zeolite crystallization during the subsequent hydrothermal crystallization process occurs in a confined space (hard-templating). A hierarchical ZSM-5 zeolite is obtained from this gel but shows a crystal morphology and textural properties very different in comparison with the dendritic zeolite samples.

Testing these zeolite samples in the aldol condensation reaction of furfural with cyclopentanone evidences the quite superior performance of the dendritic ZSM-5 zeolites. Thus, ZSM-5 (C14) and ZSM-5 (C18) exhibit significantly higher catalytic activity than ZSM-5 (C10) and ZSM-5 (C22–90) materials, being also quite more active than a commercial nanocrystalline ZSM-5 zeolite. These promising results are attributed to the occurrence of improved accessibility and balanced Brønsted/Lewis acidity in the dendritic ZSM-5 samples.

## Supporting Information Available

Figure S1: 1H-NMR of FC (a) and F2C (b) condensation adducts. Figure S2: TEM micrographs of GEL (C14) (a) and GEL (C18) samples (c). Figure S3: 29Si MAS NMR spectra of as-synthesized gels (n.q.: not quantifiable). Figure S4: XRD pattern (a), and TEM micrographs (b, c) of the ZSM-5 (C22–0) sample. Figure S5: TEM micrographs of ZSM-5 (C10) sample. Figure S6: TEM micrographs of ZSM-5 (C22) sample. Figure S7: 29Si MAS NMR spectra of as-synthesized zeolites obtained through crystallization at 150 °C (n.q.: not quantifiable). Figure S8: 27Al MAS NMR spectra of calcined zeolitic samples obtained through crystallization at 150 °C. Table S1: Physico-chemical properties of n-ZSM-5 sample, used as reference material (PDF)
