# Supplementary material for: Synthesis of Dendritic ZSM-5 Zeolite through Micellar Templating Controlled by the Amphiphilic Organosilane Chain Length
Source: Cryst Growth Des. 2023 Jun 28;23(8):5658–70. doi: 10.1021/acs.cgd.3c00326 (PMC10401572; doi:10.1021/acs.cgd.3c00326)
Supplement: Supplementary file 1 — cg3c00326_si_001.pdf [file cg3c00326_si_001.pdf]

Supporting Information

Synthesis of dendritic ZSM-5 zeolite through micellar templating controlled by the amphiphilic organosilane chain length

María del Mar Alonso-Doncel[†], *Elena A. Giner*[†], *Daniel de la Calle*[†], *Jennifer Cueto*[†], *Patricia Horcajada*[□], *Rafael A. García-Muñoz*[□] *and David P. Serrano*[*,†,□]

† Thermochemical Processes Unit, IMDEA Energy Institute, Avda. Ramón de la Sagra 3, E28935, Móstoles, Madrid, Spain.

□ Advanced Porous Materials Unit, IMDEA Energy Institute, Avda. Ramón de la Sagra 3, E28935, Móstoles, Madrid, Spain.

□ Chemical and Environmental Engineering Group, Rey Juan Carlos University, C/ Tulipán s/n, E28933, Móstoles, Madrid, Spain.

Table of contents

Figure S1. ^{1}H-NMR of FC (a) and F_2C (b) condensation adducts.

Figure S2. TEM micrographs of GEL (C14) (a) and GEL (C18) samples (c).

Figure S3. ^{29}Si MAS NMR spectra of as-synthesized gels (n.q.: not quantifiable).

Figure S4. XRD pattern (a), and TEM micrographs (b, c) of the ZSM-5 (C22 – 0) sample.

Figure S5. TEM micrographs of ZSM-5 (C10) sample.

Figure S6. TEM micrographs of ZSM-5 (C22) sample.

Figure S7. 29Si MAS NMR spectra of as-synthesized zeolites obtained through crystallization at 150 ºC (n.q.: not quantifiable).

Figure S8. ^{27}Al MAS NMR spectra of calcined zeolitic samples obtained through crystallization at 150 ºC.

Table S1. Physico-chemical properties of n-ZSM-5 sample, used as reference material.

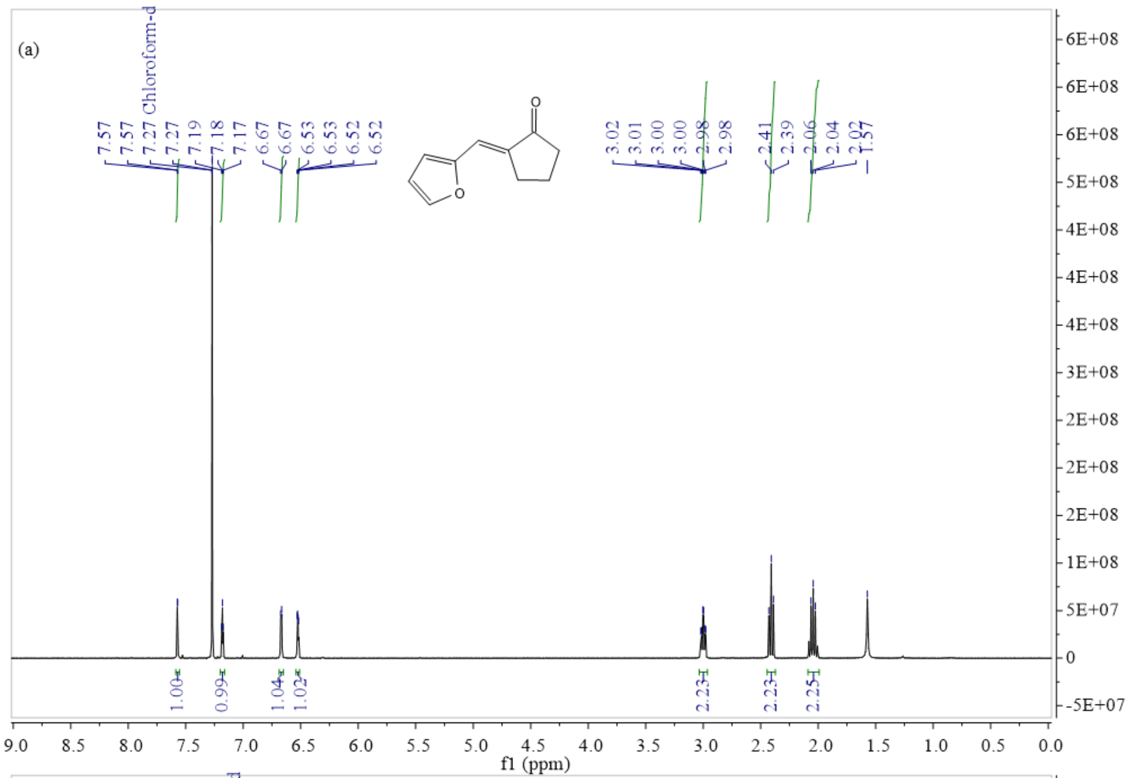

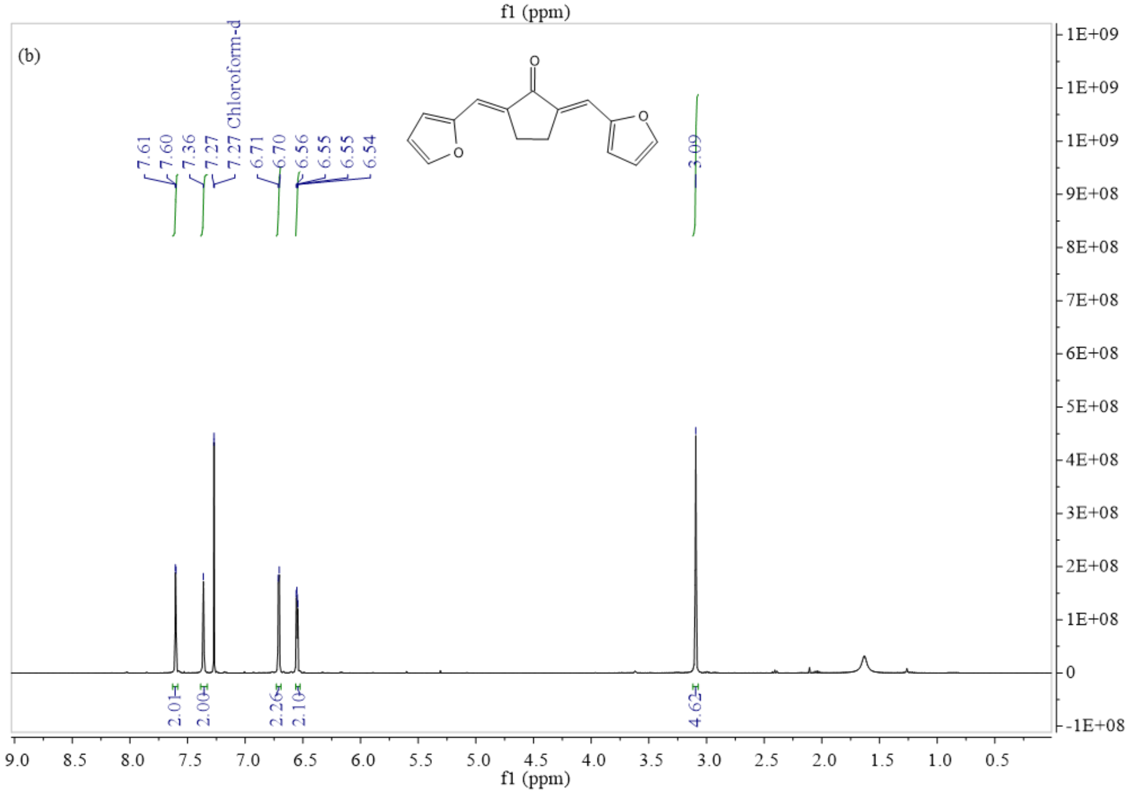

Figure S1. ^{1}H-NMR of FC (a) and F2C (b) condensation adducts.

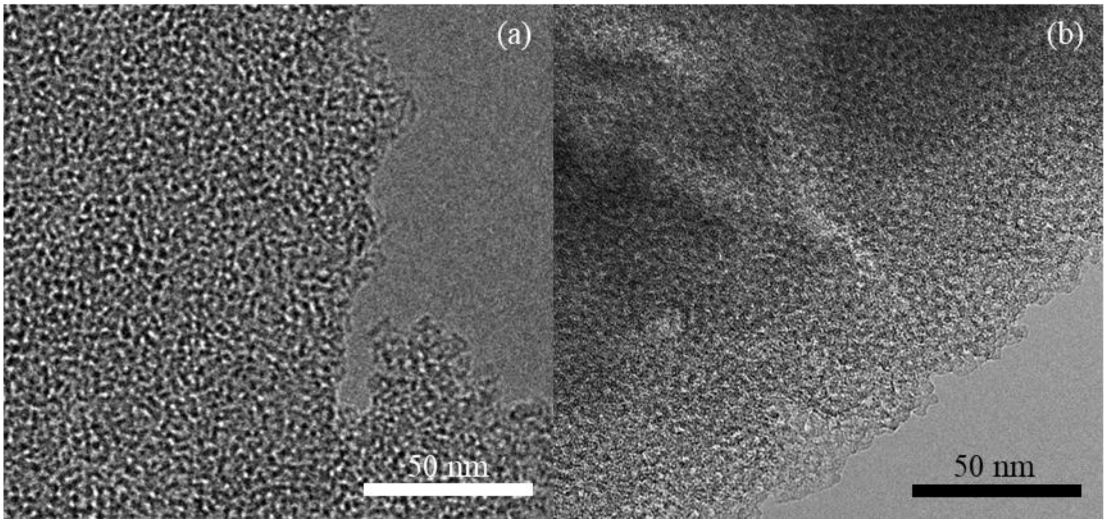

Figure S2. TEM micrographs of GEL (C14) (a) and GEL (C18) samples (b).

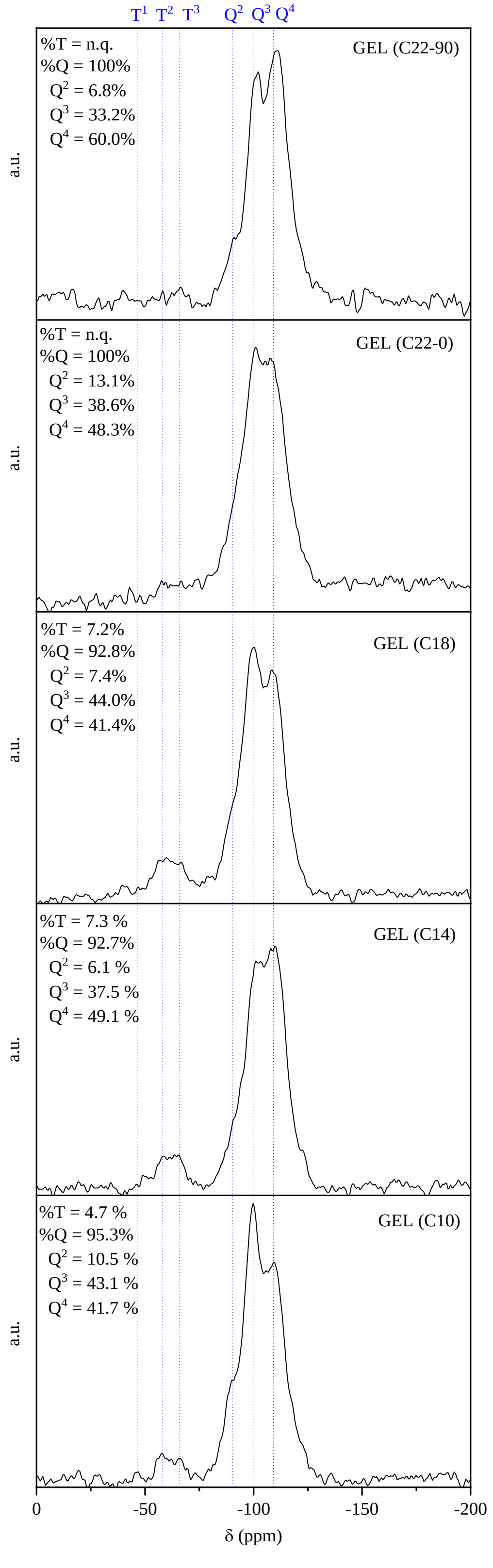

Figure S3. ^{29}Si MAS NMR spectra of as-synthesized gels (n.q.: not quantifiable).

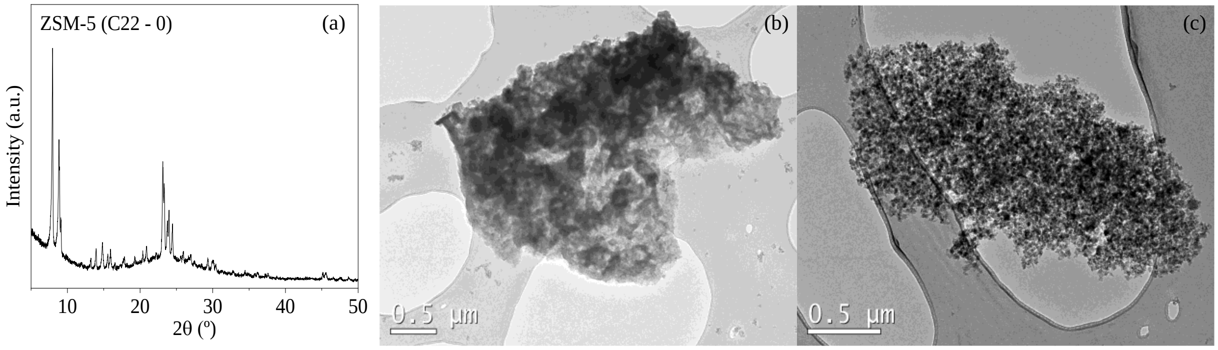

Figure S4. XRD pattern (a), and TEM micrographs (b, c) of the ZSM-5 (C22 – 0) sample.

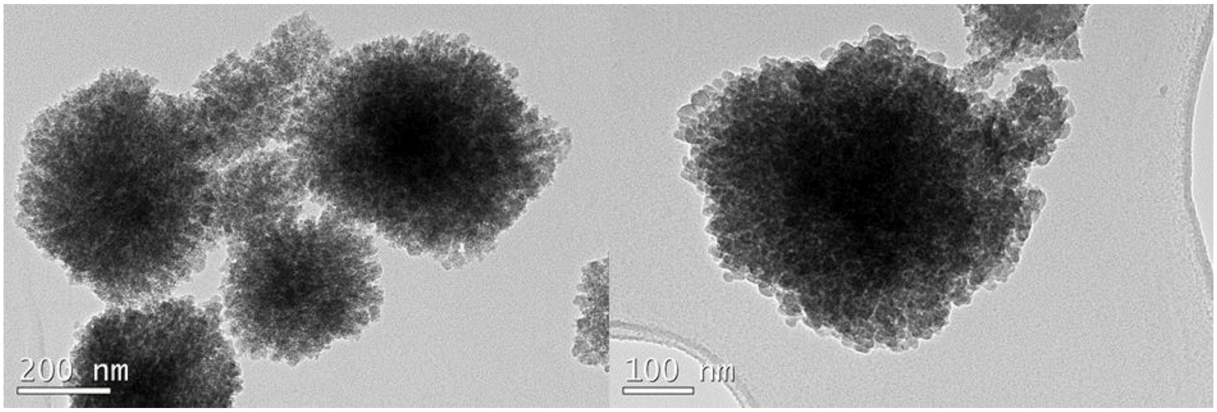

Figure S5. TEM micrographs of ZSM-5 (C10) sample.

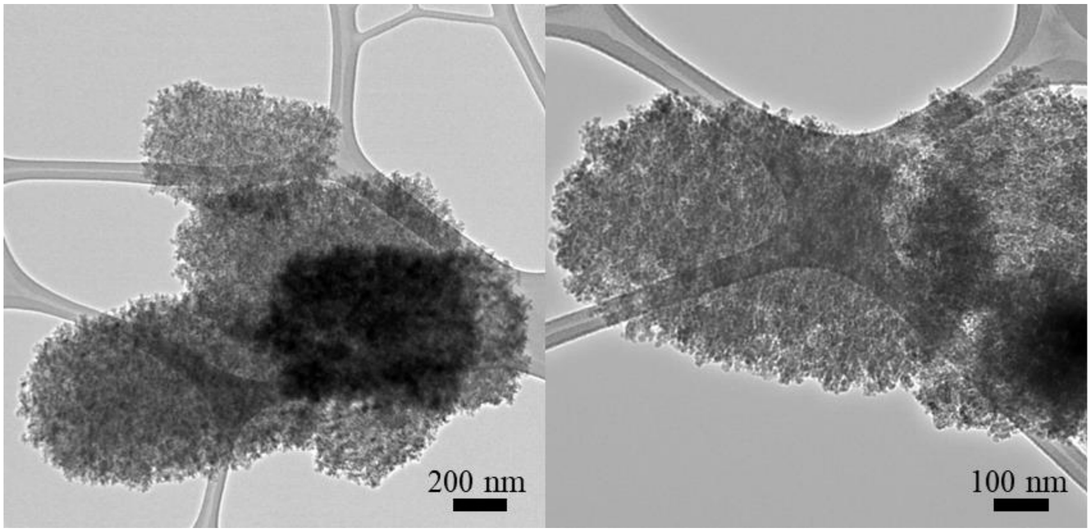

Figure S6. TEM micrographs of ZSM-5 (C22 - 90) sample.

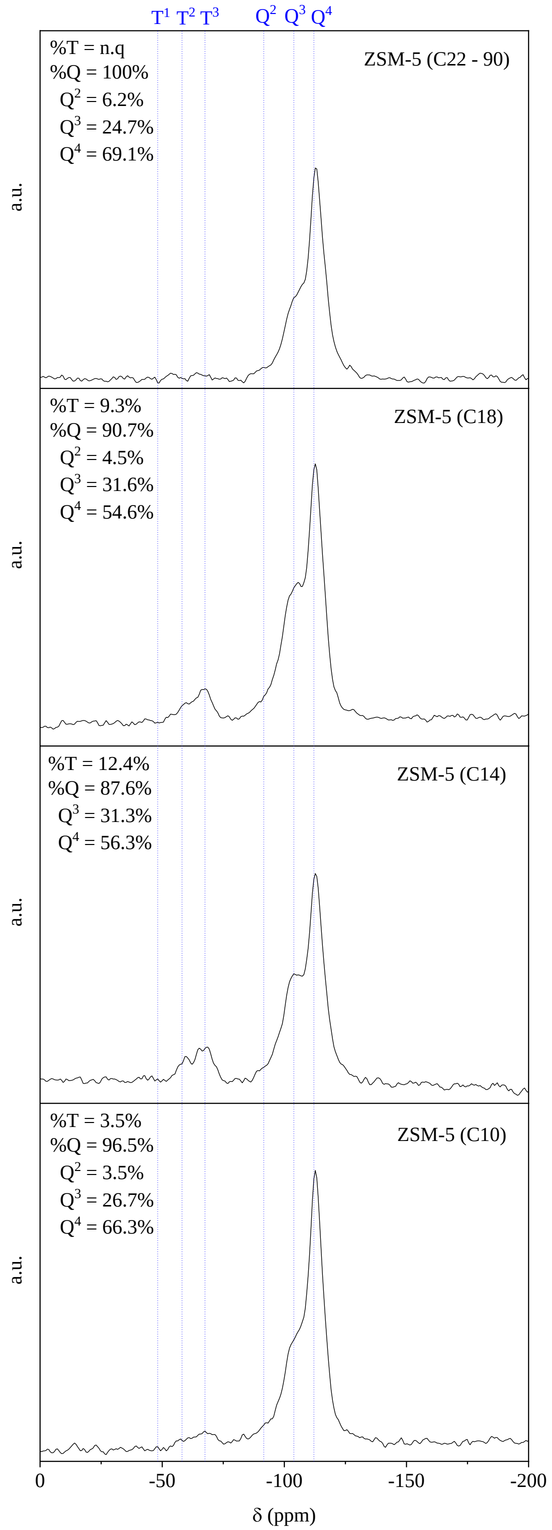

Figure S7. ^{29}Si MAS NMR spectra of as-synthesized zeolites obtained through crystallization at 150 ºC (n.q.: not quantifiable).

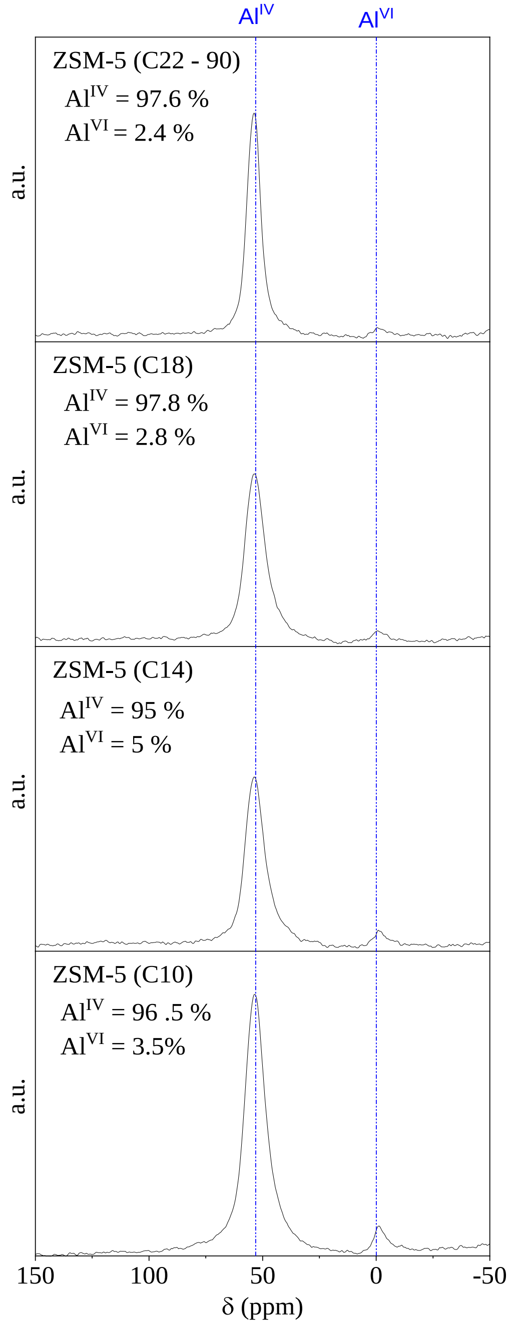

Figure S8. ^{27}Al MAS NMR spectra of calcined zeolitic samples obtained through crystallization at 150 ºC.

Table S1. Physico-chemical properties of n-ZSM-5 sample, used as reference material.

S_{BET} ($m^2 \cdot g^{-1}$)	S_{mic} ($m^2 \cdot g^{-1}$)	$S_{mes+ext}$ ($m^2 \cdot g^{-1}$)	V_T ($cm^3 \cdot g^{-1}$)	V_{mic} ($cm^3 \cdot g^{-1}$)	Si/Al	*C_B ($mmol \cdot g^{-1}$)	*C_L ($mmol \cdot g^{-1}$)	*C_B/C_L
392	275	117	0.515	0.176	42	0.205	0.091	2.25

* From FTIR-pyridine at 150 ºC.
